# Diseases of the Eye

**Published:** 1856-05

**Authors:** 


*Diseases of the Eye.*—In our coming volume we propose to devote more attention to diseases of the eye than we have heretofore bestowed. We rarely have an original contribution on any of the various subjects of ophthalmia, and shall be obliged to supply the deficiency from our own practice. Our city has outgrown its charities, and we have no eye infirmary, or kindred institution, with the exception of the private establishments of noted quacks. During the past year we have been visited by a number of these Egyptian locusts, and they have succeeded in doing an infinite amount of mischief.

Our personal experience in this branch of surgery is sufficiently large to furnish cases of interest for each number of our Journal. It is time that the profession redeemed the eye from the hands of specialists, for it, of all organs, is one which sympathises with, and partakes of, the disorders of the system at large.

